# The degree of peri-implant osteolysis induced by PEEK, CoCrMo, and HXLPE wear particles: a study based on a porous Ti6Al4V implant in a rabbit model

**DOI:** 10.1186/s13018-018-0736-y

**Published:** 2018-01-31

**Authors:** Zhe Du, Zhonglin Zhu, You Wang

**Affiliations:** 1 Department of Bone and Joint Surgery, Renji Hospital, School of Medicine, Shanghai Jiaotong University, 145 Middle Shandong Road, Shanghai, 200001 China; 2 Jiangsu OKANI Medical Technology Co., Ltd, Soochow, China

**Keywords:** Wear particles, Highly cross-linked polyethylene, Polyether-ether-ketone, Cobalt-chromium-molybdenum, Osteolysis

## Abstract

**Background:**

Polyether-ether-ketone (PEEK), cobalt-chromium-molybdenum (CoCrMo), and highly cross-linked polyethylene (HXLPE) are biomaterials used in orthopedic implants; their wear particles are considered to induce peri-implant osteolysis. We examined whether different particle types induce the same degree of peri-implant osteolysis.

**Methods:**

Forty female rabbits were randomly divided into four groups—the control group (*n* = 10), which received implantation operation and sham operation at 1 month postoperation; three experimental groups (*n* = 10 in each group), which received implantation operation along with administration of 0.1 mL of particle suspension (approximately 1.0 × 10^8^ PEEK, CoCrMo, or HXLPE wear particles) into the knee joint at 1 month postoperation. All rabbits were sacrificed at 2 months postoperation. The synovium was removed and histologically assessed. The distal femurs with the implants were analyzed via micro-computed tomography (CT) and hard tissue biopsy.

**Results:**

The average size of almost 90% of the particles was < 5 μm, indicating no significant difference in the three particle types. IL-1β, IL-8, TNFα, RANKL, and MCP-1 expression in PEEK and CoCrMo groups was high, while that in the HXLPE group was low. The bone density (BD) and bone volume/total volume (BV/TV) of the porous structures (part of the implants in all groups) in experimental groups did not decrease markedly (*p* > 0.05), while BD in the peripheral regions in experimental groups decreased markedly compared to control groups (*p* < 0.05). BV/TV in the peripheral regions was significantly decreased in PEEK and CoCrMo groups when compared to control group (*p* < 0.05), while no significant difference was noted between HXLPE and control groups (*p* > 0.05). The changes in BV observed in the hard tissue sections were consistent with those noted in the micro-CT findings.

**Conclusion:**

PEEK, CoCrMo, and HXLPE wear particles (approximately having the same size and doses) induce peri-implant osteolysis to a different degree: HXLPE particles induce peri-implant osteolysis to a mild degree, while PEEK and CoCrMo particles caused significant peri-implant osteolysis. In case of a porous implant, osteolysis occurred primarily in the peripheral region, rather than in the porous structures. Our findings would be helpful for implant designers to choose friction pairs in orthopedic components.

## Background

Polyether-ether-ketone (PEEK), cobalt-chromium-molybdenum (CoCrMo), and highly cross-linked polyethylene (HXLPE) are biomaterials commonly used in orthopedic implants. PEEK polymer has been introduced as a candidate material to be utilized for the substitution of metals in orthopedic implants [1]. Self-mating PEEK implants in the form of lumbar nucleus replacement and cervical total disc arthroplasty devices are now reported to be in clinical use [2]. Currently, metal-on-HXLPE is a popular bearing combination in total joint replacement components. For example, artificial knee joints comprise CoCrMo alloys with femoral components articulated using HXLPE on the tibial surface [3]. Furthermore, HXLPE is currently the bearing material of choice for knee arthroplasty [4, 5] as HXLPE exhibits a better wear rate than that exhibited by conventional polyethylene (PE) [6].

However, no single material is considered absolutely perfect, and generation of wear debris from any part of the prosthesis is unavoidable [7]. The hostile biological effect associated with the wear debris was first reported in 1977, which was characterized by peri-prosthetic bone loss [7, 8]. Monocyte/macrophage lineage is the major cell type involved in the wear-induced peri-prosthetic inflammatory osteolysis owing to their phagocytic role and release of pro-inflammatory mediators [7, 9, 10]. Particle characteristics (size, concentration, and composition) were a major element deciding the bio-reactivity of wear particles [11, 12]. It is reported that Ti debris is more potent than PE particles of similar sizes [11]. However, little agreement exists on the type of biomaterial debris, which are more bio-reactive, and contradictory statements were reported by some authors [13]. Therefore, this study aimed to compare the ability of osteolytic effect of the three common wear particles, especially, by introducing PEEK particles into the debate, and to investigate the initial characteristics of osteolysis based on the porous implant. It was assumed that the polymer wear particles (PEEK and HXLPE) were less bioactive than CoCrMo particles were at the same dose, and similar size and shapes.

## Methods

### Characterization of wear particles

Commercially available particles (BioEngineering Solutions, Oak Park, IL, USA) were generated from non-sterilized bulk material of a PEEK tibial tray and an HXLPE insert (Zeniva PEEK ZA-500, Chirulen HXLPE 1020X; Jiangsu Okani Medical Technology Co., Ltd., Soochow, JS, China), using proprietary techniques involving custom cryo-milling and pulverization. The CoCrMo wear particles were obtained as a gift from 3D Systems, Inc. (Rock Hill, SC, USA). The particles were sterilized by ethylene oxide (EtO) sterilization with 2000 mg/L for 1 h at 22 °C, using the AN87 Dosimeter (Warwick, UK). Particle size analysis was performed using scanning electron microscope (SEM; PSEMII Aspex, Pittsburg, PA, USA) with a liquid sample circulator and an ultrasound dispersal system. The particles, regardless of the size, were counted in at least 15 random image fields. The sizes and shapes of the particles were assessed by image analysis of the micrographs (Scion Image/NIH Image analysis software). The minimum number of particles in each sample was 450.

### Porous Ti6Al4V implant

**Fig. 1:**
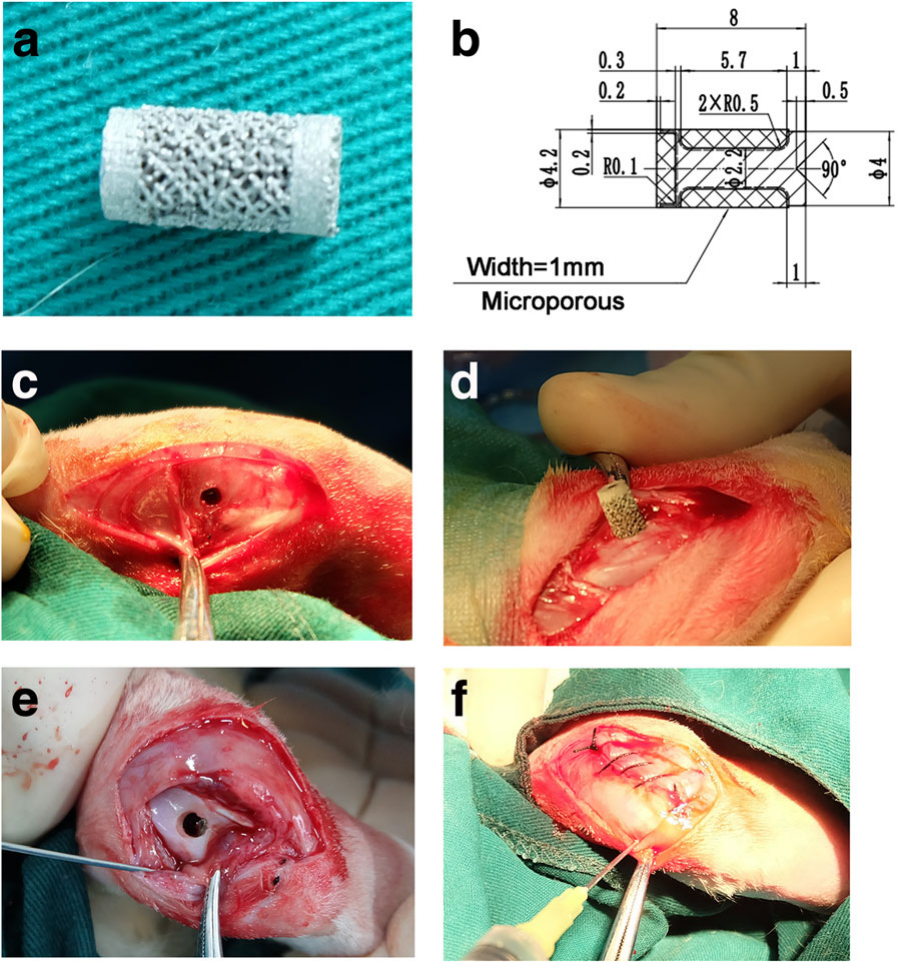
General view of implant, design parameters, and implantation process. **a** General view of implant. **b** Design parameters of the implant. **c**–**f** General implantation process

The cylindrical implant was provided by Jiangsu Okani Medical Technology (Soochow, JS, China). As shown in Fig. 1a, b, the implant was made by 3D printing with Ti6Al4V, which was 8 mm in length and 4.2 mm in width. There were two sites with 1-mm porous structures at both sides of the implants. The implant was sterilized by irradiation and packed under sterile environment.

### Animals

Forty female rabbits weighing 2.5–3.0 kg were obtained from the animal experimental center of Renji Hospital (Shanghai Jiaotong University, Shanghai, China). They were randomly assigned to one of the four treatment groups—control (*n* = 10), PEEK particles (*n* = 10), CoCrMo particles (*n* = 10), and PE particles (*n* = 10). The rabbits were kept in a surgical research institution for 1 week prior to implantation. They were kept in groups of four per cage and allowed food and water ad libitum. All the animal procedures and experiments were approved by the Animal Ethical Committee of the Renji Hospital, Shanghai Jiaotong University, School of Medicine (Shanghai, China). All experiments were performed according to the guidelines of the National Institutes of Health and the institutional rules for the use and care of laboratory animals at Shanghai Jiaotong University.

### Surgical procedures

Briefly, the rabbits were anesthetized by venous administration of ketamine (10 mg/kg). Each rabbit was immobilized with the knee joint in the maximally flexed position, and the left leg was shaved and depilated. First, a channel 15 mm in length was drilled at the end of the distal femur (just under the trochlea) using 4-mm hollow drill bits (Fig. 1c), and then, the cylindrical implant was inserted into the hole and embedded into the middle of the channel with a hammer (Fig. 1d). The wound was sutured by layers after irrigation. After surgery, the rabbits were housed in ventilated rooms with access to water and food. Then, 1 month postoperation, rabbits in the experimental groups received particle injections. A hole was drilled in the trochlea to reach the surface of the implant to build channels for particle-implant interactions (Fig. 1e). Then, the wound was tightly sutured. The three types of particle suspensions (suspension medium: phosphate buffer saline) were sonicated for at least 60 min to avoid particle aggregation prior to injection. Each particle suspension (0.1 mL; approximately 1.0 × 10^8^ wear particles) was then injected into the left knee of the experimental groups (*n* = 10 in each group) under sterile conditions (Fig. 1f). Each animal was observed and evaluated daily for general health. Rabbits in control groups received sham operations (same with experimental groups but without particle injections).

### Sample preparation

Animals were sacrificed through the intravenous injection of an overdose pentobarbital sodium 2 months after the first surgery. The synovial tissues in the knee joints and the distal femurs were harvested and fixed in 4% buffered formaldehyde for histomorphometric observation and micro-CT examination.

### Immunohistochemistry

Two sections from each knee synovial membrane were stained immunohistochemically with each of the primary antibodies (IL-1, IL-8, TNF-α, RANKL, and MCP-1 [R&D Systems, Minneapolis, MN, USA]). After staining, the two samples of each primary antibody were evaluated semi-quantitatively with a light microscope at different magnifications (× 10, × 20; Carl Zeiss, MicroImaging GmbH, Germany). The area labeled in the immunohistochemistry procedure was analyzed using Image-pro plus 6.0 (Media Cybernetics, Inc., Rockville, MD, USA). Six fields, at × 200 original magnification of each slice, were digitized and transferred to the Image-pro plus 6.0 software. The area covered by positive cells (brown color) was determined, and the brown-labeled area was then divided by the area occupied by the cells and multiplied by 100.

### Micro-CT examination

**Fig. 2:**
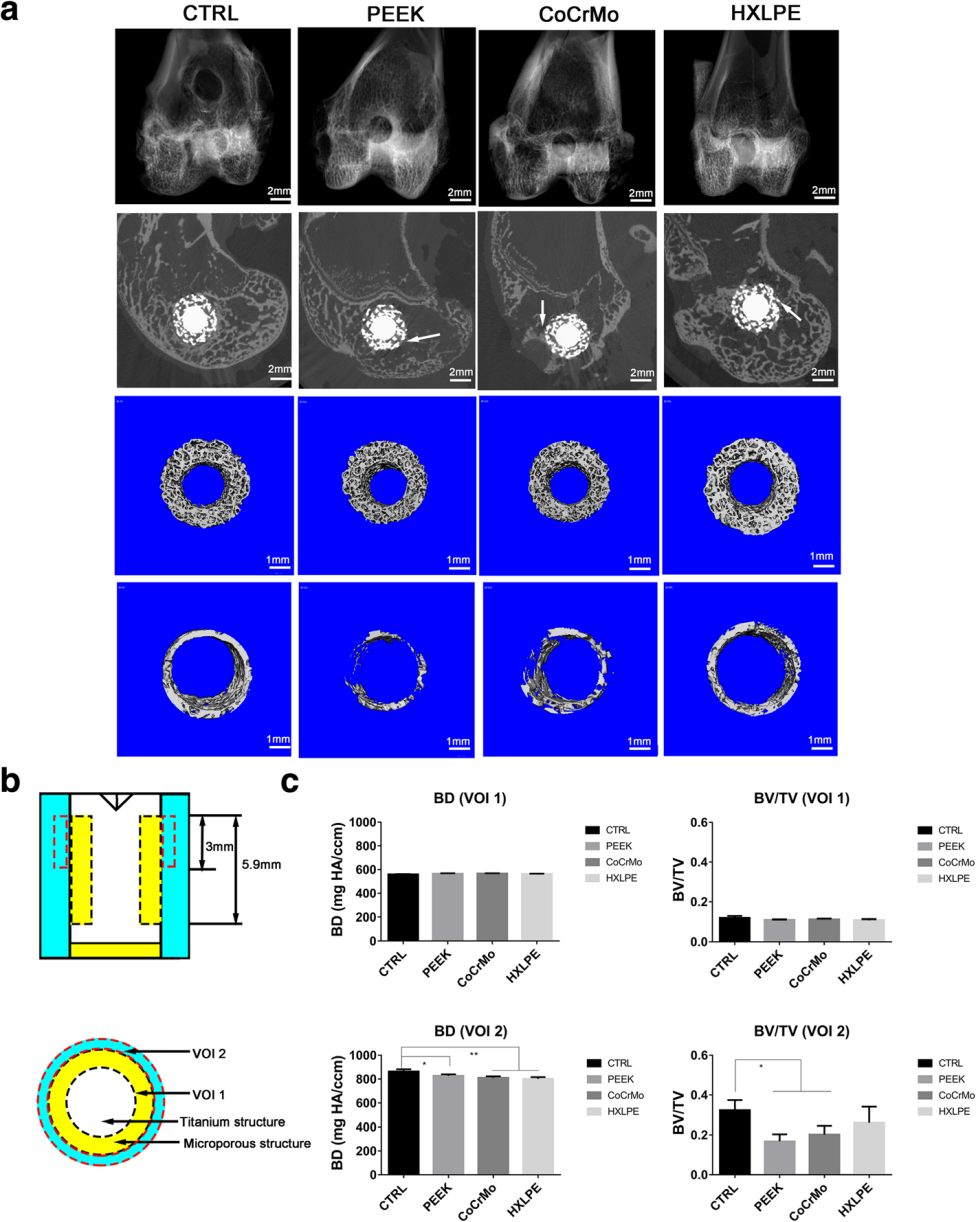
Micro-CT analysis of the distal femurs with the implants. **a** Radiological reconstruction images of the bone in the porous and peripheral regions. Plain X-ray of the distal femurs is shown in the first row. 2D reconstructions of the distal femurs are shown in the second row. 3D reconstructions of the bone in the porous and peripheral regions were, respectively, shown in the third and fourth row. **b** Diagrammatic sketch for volume of interests. **c** The results of micro-CT analysis of bone-related parameters in volume of interests. The bars represent the mean and the error bars represent the standard deviation. *p < 0.05, **p < 0.001

After the rabbits were sacrificed, the distal femurs of left hind limbs were examined using a micro-CT system (SCANCO medical AG, Barsersdorf, Zurich, Switzerland) with 30-μm axial slices. According to the recommended reporting guidelines for methodology [14], the 2D images of the distal femur of an adult rabbit were scanned at voxel size of 12 μm. Images were acquired at 70 kVp, 30 μA, and 300-ms integration time. As shown in Fig. 2b, the porous structure was defined as volume of interest 1 (VOI 1, 1 mm in width, 5.9 mm in height). The peripheral circular volume, which was 0.5 mm in diameter and 3 mm in height, was defined as volume of interest 2 (VOI 2). Bone density (BD) and bone volume/total volume (BV/TV) were analyzed in VOI 1 and 2. The 2D images of the distal femurs and 3D images of both porous (VOI 1) and peripheral (VOI 2) new bones were reconstructed.

### Hard tissue biopsy

The distal femurs were dehydrated stepwise using 70, 95, and 100% ethanol, followed by incubation in methyl methacrylate. A Leica diamond saw (Leica SP1600) was used to cut the resin blocks into 150 μm thickness, parallel to the long axis of the femoral shaft. The sections were grounded and polished to a thickness of about 50 μm. Finally, the specimens were stained using Van Gieson’s picrofuchsin. The final slices were observed using a light microscope (× 4, × 10; Carl Zeiss MicroImaging GmbH, Germany).

### Statistical analysis

Data were analyzed using one-way analysis of variance (ANOVA) with LSD post hoc *t* tests. Whitney *U* or Wilcoxon rank sum tests were used for unpaired and paired non-parametric data. Differences with *p* < 0.05 were considered statistically significant.

## Results

### Wear particle characterization

**Fig. 3:**
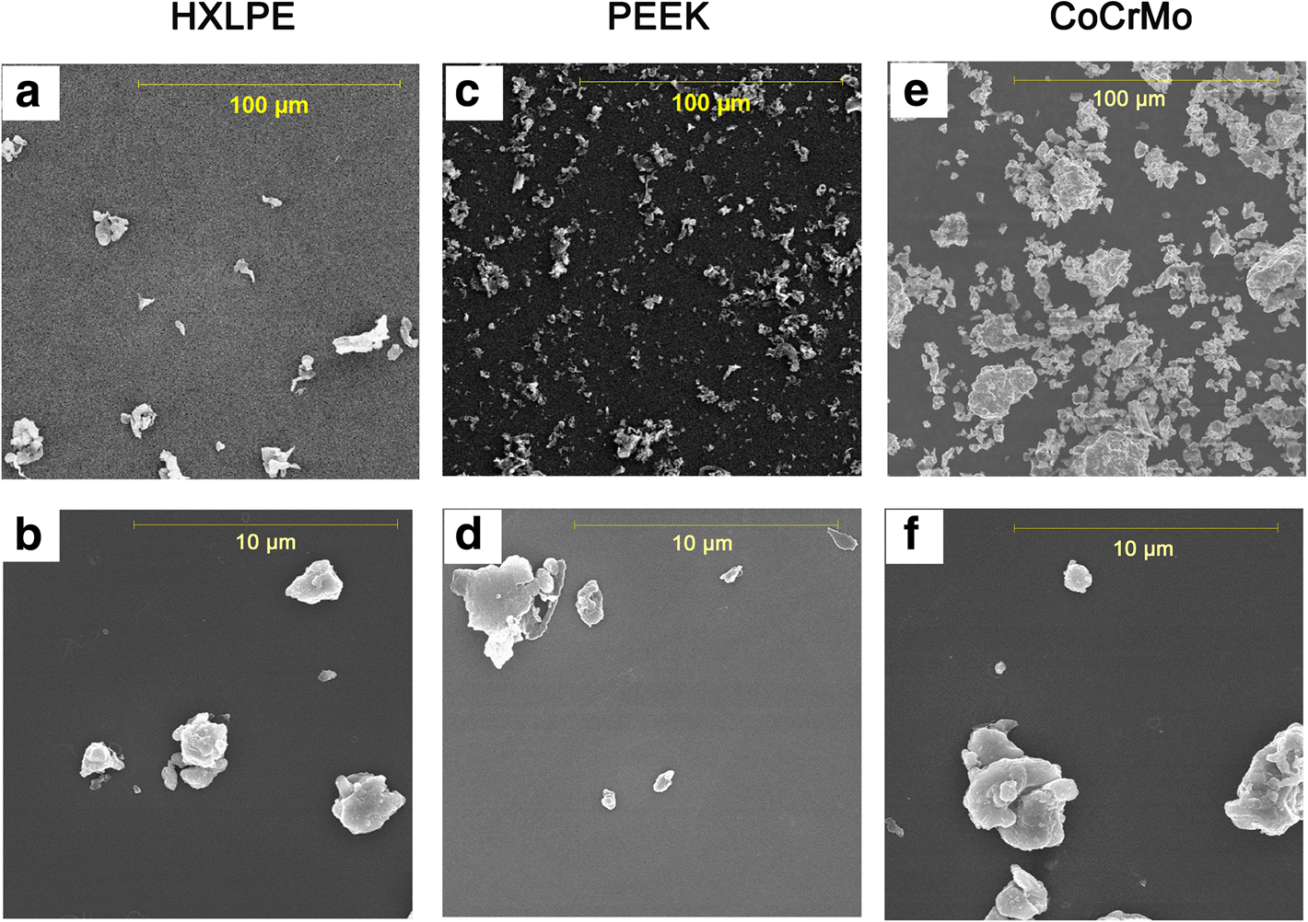
Scanning electron microscopy (SEM) images of wear debris from HXLPE, PEEK, and CoCrMo. **a**, **b** Scanning electron microscopy (SEM) images of HXLPE wear debris in two magnification fields (**a** magnification × 1000, **b** magnification × 10,000). **c**, **d** Scanning electron microscopy (SEM) images of PEEK wear debris in two magnification fields (**c** magnification × 1000, **d** magnification × 10,000). **e**, **f** Scanning electron microscopy (SEM) images of CoCrMo wear debris in two magnification fields (**e** magnification × 1000, **f** magnification × 10,000)

**Fig. 4:**
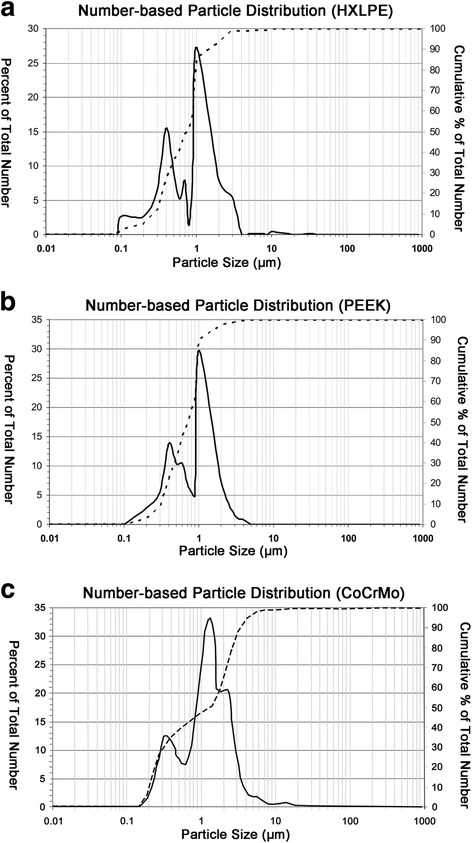
Size distribution of HXLPE, PEEK, and CoCrMo particles (number based), as determined using scanning electron microscopy. **a** Number-based particle distribution of HXLPE particles. **b** Number-based particle distribution of PEEK particles. **c** Number-based particle distribution of CoCrMo particles

The SEM micrographs showed that the particles exhibited similar morphological characteristics, i.e., globular or granular shapes, at all size ranges (Fig. 3). The mean size of the HXLPE particles was 1.23 μm in diameter. The size range was 0.16–101.72 μm, with 96% of the particles measuring less than 5 μm in diameter and 70% in the submicron range (Fig. 4a, Table 1). The mean particle size of the PEEK sample was 1.05 μm in diameter. The size range was 0.25–46.47 μm in diameter, with 99% of the particles measuring less than 5 μm in diameter and 93% in the submicron range (Fig. 4b, Table 1). The mean size of the CoCrMo particles was 1.16 μm in diameter. The size range was 0.39–61.65 μm in diameter, with 90% of the particles measuring less than 5 μm in diameter and 50% in the submicron range (Fig. 4c, Table 1). Measurements of the particle size, aspect ratio, roundness, form factor, and perimeter are shown in Table 1.

**Table 1:** SEM particle analysis of HXLPE, PEEK, and CoCrMo particles

	HXLPE	PEEK	CoCrMo
Particle size (μm)	1.23 (0.72)	1.05 (0.70)	1.16 (1.13)
Aspect ratio	1.79 (1.60)	1.83 (1.53)	1.81 (1.59)
Roundness	0.60 (0.50)	0.61 (0.52)	0.60 (0.51)
Form factor	0.66 (0.61)	0.67 (0.60)	0.67 (0.60)
Perimeter (μm)	5.62 (2.33)	4.51 (2.12)	4.96 (2.65)

Parameter values are reported as means (medians)

### Immunohistochemical examination

**Fig. 5:**
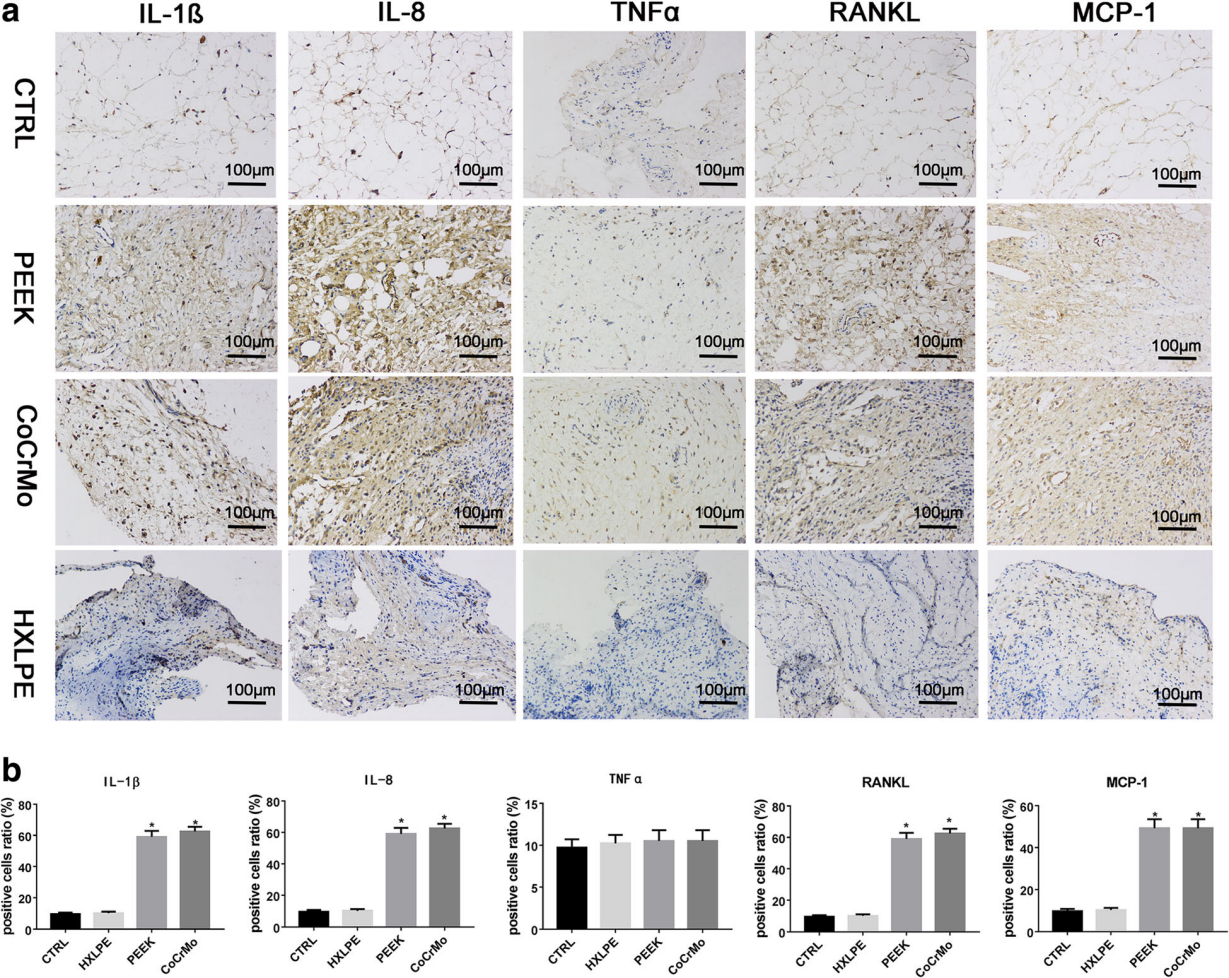
Immunohistochemical staining of the synovium. **a** Immunohistochemical staining (IL-1β, IL-8, TNFα, RANKL, MCP-1) of the synovium. **b** Semi-quantitative analysis of the positive area. The bars represent the mean, and the error bars represent the standard deviation. *p < 0.05 when compared with controls (magnification × 200, bar = 100 μm)

As the semi-analysis indicated, IL-1β, IL-8, TNFα, RANKL, and MCP-1 were strongly expressed in PEEK and CoCrMo groups and weakly expressed in the HXLPE group compared to that in the control group (Fig. 5).

### Micro-CT examination

The 2D images of the distal femurs and 3D images of both porous (VOI 1) and peripheral (VOI 2) new bones were reconstructed (Fig. 2a). The BD and BV/TV in the porous structures (VOI 1) did not decline markedly (*p* > 0.05, Fig. 2c), while BD in the peripheral regions (VOI 2) decreased markedly as compared to those in the control group (*p* = 0.03 when comparing PEEK and CTRL; *p* < 0.001 when comparing CoCrMo, HXLPE, and CTRL, Fig. 2c). BV/TV in the peripheral regions (VOI 2) decreased significantly in the PEEK and CoCrMo groups (*p* = 0.02, Fig. 2c), while no significant difference was noted between HXLPE and control groups (*p* > 0.05, Fig. 2c).

### Hard tissue biopsy

**Fig. 6:**
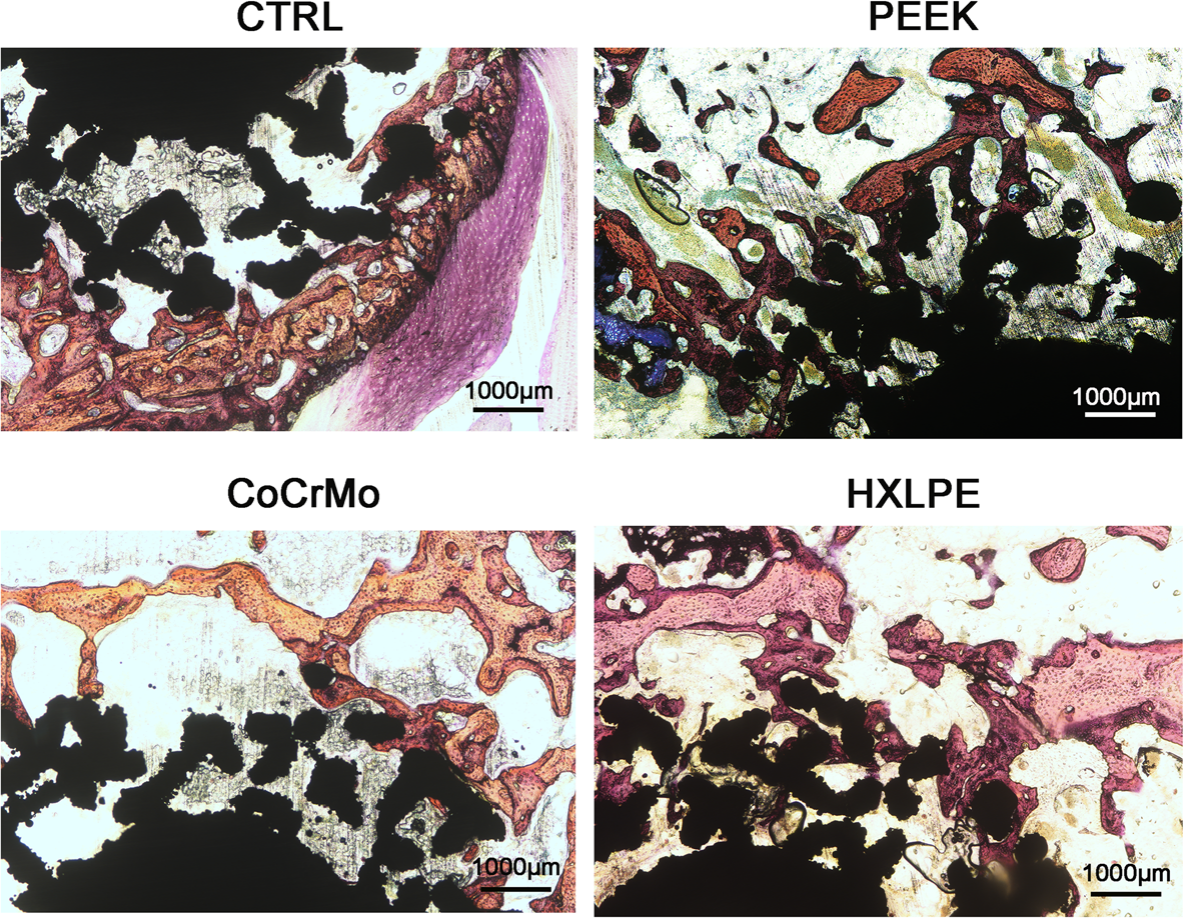
Cross-sectional images of the hard tissue sections (magnification × 40, bar = 1000 μm). The specimens were stained using Van Gieson’s picrofuchsin. The BV (red) in the peripheral regions was obviously decreased in PEEK and CoCrMo groups as compared with that in the control group, while the BV was mildly decreased in the HXLPE group. However, there were no obvious changes in BV in the porous structures among the experimental groups

The BV (red) in the peripheral regions was obviously decreased in PEEK and CoCrMo groups as compared with that in the control group, while the BV was mildly decreased in the PE group. However, there were no obvious changes in BV in the porous structures among the experimental groups (Fig. 6).

## Discussion

Peri-implant osteolysis refers to peri-prosthetic bone destruction as observed on radiographs and corresponds to bone defects. As a result, up to 15% of the patients are likely to be suffering from aseptic loosening in the decade following a total joint arthroplasty [15]. The occurrence of peri-prosthetic osteolysis is closely associated with the particle type, size, volume, and especially the functional biological activity (FBA) of wear debris. FBA of wear debris is calculated using the following formula: FBA = *V* × SBA (where *V* is the volumetric wear rate and SBA is the specific biological activity) [16]. Comprehensive understanding about the intricate process of osteolysis is of utmost importance for future development of therapeutic modalities that may delay or prevent the disease progression. Therefore, this study investigated the bioactivity of inducing peri-implant osteolysis among three bearing wear particles and found their effects on porous implant.

It is known, based on in vitro testing, that a particle should have a phagocytosable size to induce an inflammatory reaction (< 10 μm), with 0.24–7.2-μm size range being the most pro-inflammatory [7]. In this study, the average size of almost 90% of the wear particles was < 5.0 μm in diameter, and the particles were in similar shapes. Macrophages play a pivotal role in wear particle recognition and in the cascade of biological events leading to implant failure. The interaction of macrophages with wear debris triggers the release of pro-inflammatory factors, such as TNF-α and IL-1; pro-osteoclastic factors, such as RANKL (receptor activator of nuclear factor κΒ ligand); and chemokines, such as MCP-1 (monocyte chemotactic protein-1), all being crucial to the recruitment, migration, differentiation, and ultimate activation of bone-resorbing osteoclasts [15]. IL-1 possesses multiple and diverse properties, especially mediating the acute phase response to endogenous and exogenous stimuli acting on macrophages [17, 18]. Shanbhag et al. [19] found that IL-6 and IL-8 could be the primary drivers of end-stage osteolysis, as opposed to TNF-α and IL-1β. RANKL is a receptor ligand expressed on the cell surface of osteoblasts, which is the key factor regulating the differentiation and activation of osteoclasts [15, 20]. MCP-1, also known as CCL2 (CC chemokine ligand-2), can attract macrophages to the sites of inflammation through the activation of CCR2 (CC chemokine receptor-2) or CCR4 (CC chemokine receptor-4). Furthermore, wear particles stimulate chronic inflammation and bone destruction that may ultimately result in implant loosening [15]. In this study, the immunohistochemical analysis of synovial tissues revealed significant expression of IL-1, IL-8, TNF-α, RANKL, and MCP-1 in the PEEK and CoCrMo groups. However, the above indicators were mildly expressed in the HXLPE group. This indicated that PEEK and CoCrMo wear particles were more bioactive in the induction of peri-implant osteolysis compared to HXLPE wear particles.

The results of micro-CT and hard tissue sections showed that PEEK and CoCrMo wear particles induced more severe osteolysis in the peripheral regions around the implant, while HXLPE wear particles induced mild osteolysis. The results were also verified by immunohistochemical analysis of the synovial tissues as discussed above. Further, the initial osteolysis occurred in the peripheral regions rather than in the porous structures. These findings overturned the original hypothesis that the polymer wear particles (PEEK and HXLPE) were less bioactive than CoCrMo particles, as the findings herein suggest that PEEK particles may be just as bioactive as CoCrMo.

PEEK has become highly attractive for use as a biomaterial for trauma and orthopedic applications, and it has already been successfully employed for spinal surgery [21, 22]. In addition, a recent study revealed the potential of PEEK as a surface material for artificial joints along with HXLPE as the other articulating surface [3]. This study verified the feasibility of PEEK, as it did not show higher bioactivity (SBA) than the currently used CoCrMo in total joint replacement. Our preliminary studies demonstrated that the HXLPE volumetric wear rate of the PEEK-on-HXLPE bearings was lesser than that of the CoCrMo-on-HXLPE bearings (unpublished data). Further, theoretically, FBA of HXLPE in PEEK-on-HXLPE bearings would be considerably less (assuming that the PEEK and CoCrMo volumetric wear rates are negligible). Thus, using PEEK, instead of CoCrMo, as the bearing surface against HXLPE will reduce the loosening of artificial joints in the long run. In addition, there has been a growing interest in the use of PEEK as a bearing material instead of HXLPE that is currently used in total joint arthroplasty. This would further require preparation of CoCrMo-on-PEEK as the bearing surface [5]. However, the findings of this study suggested that at similar doses and sizes, both CoCrMo and PEEK wear particles resulted in osteolysis. Further, pin-on-plate tests performed on unfilled PEEK against CoCrMo displayed high wear rates for PEEK [23]. Therefore, we do not recommend using PEEK instead of HXLPE and CoCrMo-on-PEEK as a friction pair.

This study has some limitations. First, the wear condition alone may not represent the nature of the wear in clinical cases; thus, further studies using wear particles isolated from tissues or validated joint replacement simulators, instead of manufactured particles, are required. Second, nanosized wear particles have been identified both in in vitro wear test lubricants and in tissues retrieved during revision surgery; our study did not include nanosized wear particles, which need to be discussed in future studies. Third, the observation time in this study was relatively short; longer observation periods are needed to find osteolysis features in the porous implant.

## Conclusions

In summary, PEEK, CoCrMo, and HXLPE wear particles (approximately with same size and dose) induce peri-implant osteolysis to different degrees: HXLPE particles induced peri-implant osteolysis to a mild degree, while PEEK and CoCrMo particles caused significant peri-implant osteolysis. Furthermore, osteolysis occurred primarily in the peripheral implant, rather than in the porous structures of the implant. Our findings would be helpful for implant designers to choose friction pairs for orthopedic components.

## Abbreviations

BD: Bone density
BV/TV: Bone volume/total volume
CCR2: CC chemokine receptor-2
CCR4: CC chemokine receptor-4
CoCrMo: Cobalt-chromium-molybdenum
HXLPE: Highly cross-linked polyethylene
MCP-1: Monocyte chemotactic protein-1
PE: Polyethylene
PEEK: Polyether-ether-ketone
RANKL: Receptor activator of nuclear factor κΒ ligand
SEM: Scanning electron microscope
